# Effects of Phytosterols on Growth Performance, Serum Indexes, and Fecal Microbiota in Finishing Pigs

**DOI:** 10.3390/ani15091188

**Published:** 2025-04-22

**Authors:** Renjie Xie, Zhenxing Guo, Haiqing Gan, Dexing Hou, Guang Chen, Chao Deng, Hongkun Li, Jiajie Ouyang, Qiyu Tian, Xingguo Huang

**Affiliations:** 1 College of Animal Science and Technology, Hunan Agricultural University, Changsha 410128, China; xrj101900@foxmail.com (R.X.); gzx247499@163.com (Z.G.);; 2 Yuelushan Laboratory, Changsha 410128, China; 3 Graduate School of Agriculture, Forestry and Fisheries, Kagoshima University, Kagoshima 890-0065, Japan; 4 Hunan Biological and Electromechanical Polytechnic, Changsha 210127, China

**Keywords:** phytosterols, fattening pigs, apparent digestibility, serum parameters, antioxidant capacity, fecal flora

## Abstract

Phytosterols (PSs) are widely present in plants, particularly abundant in plant oils and seeds. PSs are reported to have various biological activities, such as lowering cholesterol, alongside antioxidant and antibacterial activities. This research examined the effects of PSs in finishing pigs, including growth performance, serum biochemistry, and fecal bacteria. Two treatment groups (each treatment group consisted of five biological replicates, and each replicate comprised five pigs housed communally) were randomly assigned to the fifty finishing pigs (equally divided by sex) of “Duroc × Landrace × Yorkshire” three-way cross with 79.76 ± 1.29 (kg) body weight. The control group (CON) was given basic food, while the experimental group was given basic food containing 300 mg PS/kg (PS). Dietary PS supplementation markedly raised the levels of average daily feed intake (ADFI) and apparent digestibility of dry matter (DM), crude protein (CP), ether extract (EE), and gross energy (GE) in comparison to the CON (*p* < 0.05). Additionally, PSs also significantly boosted the concentrations of high-density lipoprotein cholesterol (HDL-C), total protein (TP), catalase (CAT), superoxide dismutase (SOD), total antioxidant capacity (T-AOC), immunoglobulin G (IgG), motilin (MTL), and glucagon-like peptide-1 (GLP-1) (*p* < 0.05), and lowered the lactate dehydrogenase (LDH) level (*p* < 0.05). Both at the phyla and genus levels, the relative abundance of Firmicutes and *Streptococcus* increased significantly, and the relative abundance of *Acinetobacter* decreased significantly when adding phytosterols (*p* < 0.05). Overall, phytosterols dietary supplementation promotes immunity and antioxidant capacity in finishing pigs and boosts growth performance by improving nutrient digestibility.

## Simple Summary

Phytosterols, as a natural plant extract, have demonstrated efficacy in improving diabetes, lowering cholesterol levels, anti-inflammation, inhibiting bacteria, anti-oxidation, modulating immune responses, preventing cardiovascular disease, promoting wound healing, enhancing capillary circulation, and promoting animal growth. The results of this study indicate that phytosterols have a positive effect on nutrient digestion, oxidative stress and immunity in finishing pigs, and phytosterols may play a positive role by regulating fecal microbiota.

## 1. Introduction

Phytosterols (PS) are bioactive components with a molecular structure similar to that of animal sterols [1]. As a plant-derived steroid, PSs are widely found in the roots and stems of plants in nature, which are particularly abundant in plant seeds and vegetable oils, such as soybean, corn, and flaxseed [2,3]. PSs have a long history of development and application. PSs are widely used in food industry, pharmaceuticals, health care, cosmetics, and other related fields [4]. With the development of plant extraction technology [1], PS, as a natural plant extract [5], have demonstrated efficacy in improving diabetes, lowering cholesterol levels, anti-inflammation, inhibiting bacteria, anti-oxidation, modulating immune responses, preventing cardiovascular disease, promoting wound healing, enhancing capillary circulation, and promoting animal growth [6]. It is reported that PS achieves the body’s anti-inflammatory effects by inhibiting cyclooxygenase, antagonizing transient Receptor Potential Vanilloid 1 (TRPV1) receptors, and attenuating proinflammatory cytokines and related mediators [7]. A mixture containing α-spinasterol showed improved antioxidant activity even at low doses (0.8 μg/mL) [8]. Additionally, PSs may improve inflammatory responses via controlling the expression of the Liver X Receptor-α (LXR-α) and Liver X Receptor-β (LXR-β), two nuclear receptors, and their downstream ATP-Binding Cassette Transporter A1 (ABCA1) and ATP-Binding Cassette Transporter G1 (ABCG1), within murine microglial cell line BV2 microglia [9]. Moreover, PSs isolated from neem leaves and Albizzia julibrissin bark had in vitro antibacterial activity against *Bacillus subtilis*, *Staphylococcus aureus*, *Pseudomonas aeruginosa,* and *Escherichia coli* [10]. In previous animal studies, Feng et al. showed that basal diet supplemented with 25 mg/kg phytosterols to yellow-feather broilers reduced the diversity of pathogenic flora in the cecum contents to a certain extent [11]. Hu et al. showed that the addition of 0.02% phytosterols to diets significantly reduced serum total cholesterol in piglets, while diarrhea rates decreased [12]. These studies have pointed out that PS, as a natural plant additive, has shown great potential in livestock and poultry feed applications, nutrition, and health research [13,14,15,16]. The ban on antibiotics has made it urgent to find a new natural plant-based feed additive to optimize the growth performance and immune system of finishing pigs [17,18]. We believe that phytosterols may enhance growth, antioxidant capacity and immunity in pigs. Based on the safety [19,20] and bioavailability advantages of phytosterols [21,22], this research examined the effects of PSs on growth performance, digestive performance, and serum indicators, as well as intestinal health.

## 2. Materials and Methods

### 2.1. Ethical Approval

The animal experiment in this research adhered to the guidelines set by the Hunan Agricultural University Institutional Animal Care and Use Committee, following their established protocols for animal welfare and experimental procedures (Permission No. 2020041).

### 2.2. Experimental Design

Hunan Heyiyuan Biotechnology Co., Ltd. (Changsha, China). supplied the phytosterols (PSs) for this trial. The phytosterols product was extracted from soybeans. Analytical assays revealed a purity of 95.26% for the sample utilized in this study—it mainly consisted of 50.93% β-sitosterol, 29.93% campesterol, 18.26% stigmasterol, and 0.98% brassicasterol.

The animal experiments were conducted at the Kelikang Experimental Base in Liuyang. Fifty healthy, similar-weight (79.76 ± 1.29 kg) “Duroc × Landrace × Yorkshire” pigs were used (Changsha Kelikang Agriculture and Animal Husbandry Technology Co., Ltd., Changsha, China, supplied the test subjects). We randomly sorted 50 pigs into two treatment groups. Each group consisted of five biological replicates, and each replicate comprised five pigs housed communally. The control group (CON) received a standard basal diet. The experimental group (PS), on the other hand, was given the same basal diet but with a phytosterol (PS) supplement added at a rate of 300 mg/kg. PSs were mixed evenly with the basal diet using a feed mixer. The CON group and the PS group were provided with a standard diet throughout a 7-day pre-feeding phase. Following this, the experimental period lasted for 35 days. The diet ingredients of this research were tailored to meet the nutritional needs of finishing pigs, as outlined by the National Research Committee (NRC, 2012, https://nap.nationalacademies.org/catalog/13298/, accessed on 16 March 2025) (Table 1). Feed was provided twice a day at 8:00 and 17:00, providing unrestricted access to food and water. The pigs’ consumption and health status were monitored and documented.

### 2.3. Record of Growth Performance

At day 0 and 35 days later, we weighed the experimental pigs in each biological replicate after a 12 h overnight fast to obtain their fasting body weights. The feed intake of the pigs was recorded for each biological replicate during the experiment. On this basis, the average daily feed consumption (ADFI), average daily weight gain (ADG), and feed efficiency ratio (F:G) were calculated.

### 2.4. Sample Collection

During days 32 to 35 of the experiment, we collected fresh fecal samples from each replicate twice a day, in the morning and in the afternoon, and gave them a good mix. After that, they were transferred into sample bags and 15 mL Eppendorf tubes and stored at −20 °C and −80 °C, respectively, until we were ready to perform the tests. As for the feed samples, we collected those weekly from both the control (CON) and PS group, mixed them up thoroughly, and stashed them away in a −20 °C freezer, pending analysis.

For 10 h, from the evening of day 34 to the morning of day 35, the experimental pigs were fasted, but allowed free access to water. From each biological replicate, we randomly picked one healthy pig. Once the feeding ceased, we drew blood from the anterior vena cava of these pigs (5 mL of vacutainers produced by Shandong Yongkang Medical Products Co., Heze, China). Roughly 10 mL of whole blood was collected from each pig’s anterior vena cava into vacutainers. After letting the samples sit at room temperature for half an hour, we spun them down at 3500 rpm for 10 min. The resulting supernatant serum was then carefully extracted and stored in a −80 °C freezer, ready for analysis.

### 2.5. Measurement of Nutrient Apparent Digestibility

Feed and fecal specimens underwent desiccation at 65 °C for durations of 12 h and 72 h, and then remoistened at room temperature overnight. They were crushed into powder and sieved through a 40-mesh sieve for collection and testing. The samples were dried in a thermostat set to 105 °C. Once a constant weight was achieved, the samples were weighed to document the dry matter (DM) weight. Sample composition regarding crude protein (CP), crude fiber (CF), and ether extract (EE) was analyzed. An SDAC-6000 automatic calorimeter (Hunan Sundy Technology Co., Ltd., Changsha, China) measured gross energy (GE). The apparent digestibility of nutrients was assessed using the endogenous indicator method using acid-insoluble ash (AIA) as an endogenous indicator.

### 2.6. Measurement of Serum Biochemical Indices

A comprehensive panel of serum biomarkers was analyzed utilizing a fully automated biochemistry analyzer (Excellence-450, Shanghai Kehua Experimental System Co., Ltd., Shanghai, China). Specifically, we assessed the levels of alanine aminotransferase (ALT), alkaline phosphatase (ALP), glucose (GLU), blood urea nitrogen (BUN), total cholesterol (TC), aspartate aminotransferase (AST), low-density lipoprotein cholesterol (LDL-C), triglycerides (TG), albumin (ALB), globulin (GLB), lactate dehydrogenase (LDH), high-density lipoprotein cholesterol (HDL-C), and total protein (TP). The detection kit used was purchased from the Nanjing Jiancheng Bioengineering Institute (Nanjing, China).

### 2.7. Measurement of Serum Antioxidant Indexes, Immune Cytokines, Hormone Level

We assessed serum levels of glutathione peroxidase (GSH-Px), total antioxidant capacity (T-AOC), superoxide dismutase (SOD), glutathione (GSH), catalase (CAT), and malondialdehyde (MDA). To measure these, we used commercially available kits sourced from the Nanjing Jiancheng Bioengineering Institute. The measurements themselves were taken using an Infinite-200 PRO multifunctional microplate reader, manufactured by Tecan Austria GmbH, Grödig, Austria.

Immunoglobulin A (IgA), immunoglobulin G (IgG), and immunoglobulin M (IgM) were measured using ELISA kits obtained from Hunan Aifang Biotechnology Co., Ltd. (Changsha, China).

Serum motilin (MTL), gastrin (GAS), and glucagon-like peptide-1 (GLP-1) levels were quantified via ELISA (Shanghai Zhuocai Biotechnology Co., Ltd., Shanghai, China).

### 2.8. Characterization of the Fecal Microbial 16S rRNA Gene Sequencing

During the last 3 days of the experiment, about 10 g of fresh fecal samples were collected from each biological replicate of the two groups into centrifuge tubes and stored at −80 °C. Shanghai Personal Biotechnology Co., Ltd. (Shanghai, China) was commissioned to detect 16SrRNA sequencing. The V3-V4 hypervariable region of the 16SrRNA gene (F: 5′-ACTCCTACGGGAGGCAGCA-3′, R: 5′-GGACTACHVGGGTWTCTAAT-3′) was selected for PCR amplification of fecal samples [23]. Following PCR, the resulting products underwent sequencing using the Illumina Miseq platform (QIIME2 (2019.4)). The subsequent sequencing data were then scrubbed clean of noise using the DADA2 method within the QIIME2 (2019.4) software package. We defined each unique, quality-controlled sequence as an Amplicon Sequence Variant (ASV). To generate an ASV abundance table, we normalized the data, setting the sequencing depth at 95% of the minimum sequence count across all samples. Venn diagrams, α diversity, β diversity, and other analyses of microorganisms in feces were performed based on the leveled or unleveled ASV abundance table.

### 2.9. Statistics and Data Analysis

The data are expressed as means ± standard error of the mean (SEM). Statistical analyses were performed on all data using Student’s *t*-tests in SPSS software version 24.0 (IBM SPSS, 2016, Chicago, IL, USA). We considered variances statistically significant if *p* < 0.05, and identified trends at *p* < 0.1.

To examine the relationships between gut flora biomarkers, apparent nutrient digestibility, and serum indices, Spearman correlation was carried out using the platform provided by Shanghai Personal Biotechnology Co., Ltd. (Shanghai, China).

## 3. Results

### 3.1. Growth Performance

The changes in body weight and ADFI of the two groups of finishing pigs are presented in Table 2. The InitialBW, FinalBW, ADG, and F:G did not differ significantly between CON and PS groups. Yet, dietary supplementation with PSs markedly increased the ADFI of finishing pigs compared with the CON group (*p* < 0.05).

### 3.2. Apparent Digestibility

In comparison to the CON group, PS supplementation considerably raised the apparent digestion of DM and GE in Table 3 (*p* < 0.01). Meanwhile, PSs dramatically raised CP and EE’s apparent digestibility (*p* < 0.05). Nevertheless, there was no difference in CF’s apparent digestibility between CON and PS groups.

### 3.3. Serum Biochemical Indicators

Table 4 presents the serum biochemical parameters of finishing pigs from the CON and PS groups. The two groups’ levels of ALT, ALP, GLU, BUN, TC, AST, LDL-C, TG, ALB, GLB, and other indicators did not differ significantly. However, compared with the CON, the LDH level was significantly lowered in the PS group. Conversely, dietary PS supplementation significantly elevated serum HDL-C and TP levels (*p* < 0.05).

### 3.4. Antioxidant Capacity

To evaluate the impact of dietary PS supplementation on the antioxidant capacity, oxidative stress markers including GSH-Px, MDA, GSH, CAT, SOD, and T-AOC were measured (Table 5). When the group where PSs were added was compared to the CON group, there was a rise with both SOD and CAT enzyme activity (*p* < 0.01). Similarly, T-AOC activity level was noticeably elevated in the PS group (*p* < 0.05). However, there was no difference in the activities of GSH-Px, MDA, and GSH between the CON and PS groups.

### 3.5. Serum Immune Production

Despite PS intervention, the concentrations of immune cytokines (IL-2, IL-4, IL-6, TNF-α, IgA, IgM) did not change, as indicated in Table 6. When dietary PS supplementation was administered to finishing pigs in PS versus CON, IgG serum levels were markedly higher (*p* < 0.05).

### 3.6. Gastrointestinal Hormones

To investigate the effects of PSs on gastrointestinal digestive hormones secretion, blood hormone markers as GAS, MTL, and GLP-1 were evaluated (Table 7). MTL and GLP-1 levels were significantly increased in the PS group (*p* < 0.01). No notable GAS-level variance existed across CON and PS groups.

### 3.7. Fecal Microbiota

**Figure 1:**
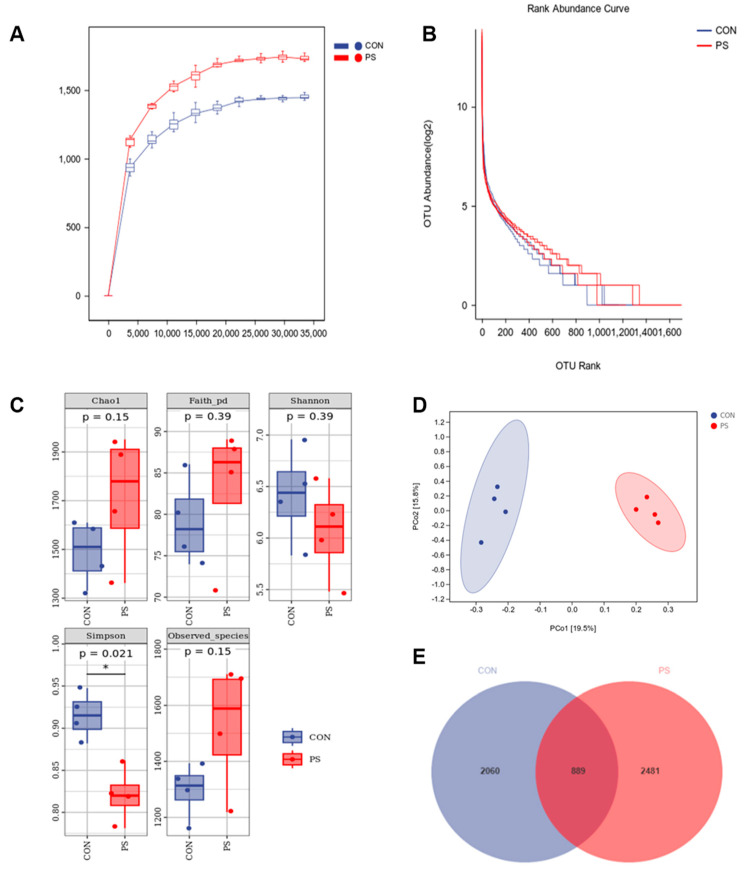
Effects of phytosterols (PSs) on diversity of fecal microbiota of finishing pigs. (**A**) Rarefaction curves illustrating microbial richness. (**B**) Rank abundance curves depicting species distribution. (**C**) α-diversity metrics, including Chao1, Faith-pd, Shannon, Simpson, and observed-species indexes. (**D**) Principal coordinate analysis (PCoA) visualizing overall community structure. (**E**) Venn diagram displaying shared and unique amplicon sequence variants (ASVs). The CON group was given a standard diet, while the PS group was offered a diet fortified with 300 mg/kg of phytosterols (PSs). Data are presented as means ± SEM (*n* = 4). * indicates *p* < 0.05.

This data quality was evaluated. The rarefaction curves stabilized, reflecting the adequacy of the sequencing depth for both the CON and PS groups (Figure 1A). Richness and homogeneity between groups were assessed using abundance rank curves (Figure 1B).

Next, variances in diversity of fecal microbial community were evaluated through α-diversity analysis, such as Chao1, Faith-pd, Shannon, Simpson and observed-species indices (Figure 1C). The observed-species, Faith-pd, and Chao1 indices increased in the PS group when compared to the CON group, while the Shannon index decreased numerically. The Simpson index of the PS group was considerably lower than that of CON (*p* < 0.05), suggesting that the PS group had more non-dominant bacterial species and a more diverse microbiological community.

β-diversity analysis was performed, as Principal Coordinates Analysis (PCoA) focuses on the differences between individual samples. As shown in Figure 1D, the primary and secondary components accounted for 19.5% and 15.8% of the CON versus PS group variance, respectively. The PCoA plot clearly separated the samples from the CON and PS groups. In both groups, 6319 Amplicon Sequence Variants (ASVs) were found, as shown in the ASV Venn diagram (Figure 1E). Specifically, the CON group contained 2949 ASVs, of which 2060 were unique to the CON group, accounting for approximately 32.6%. The PS group presented 3370 ASVs, with 2481 unique to the PS group, accounting for approximately 39.3%. A total of 889 ASVs were shared between the two groups.

**Figure 2:**
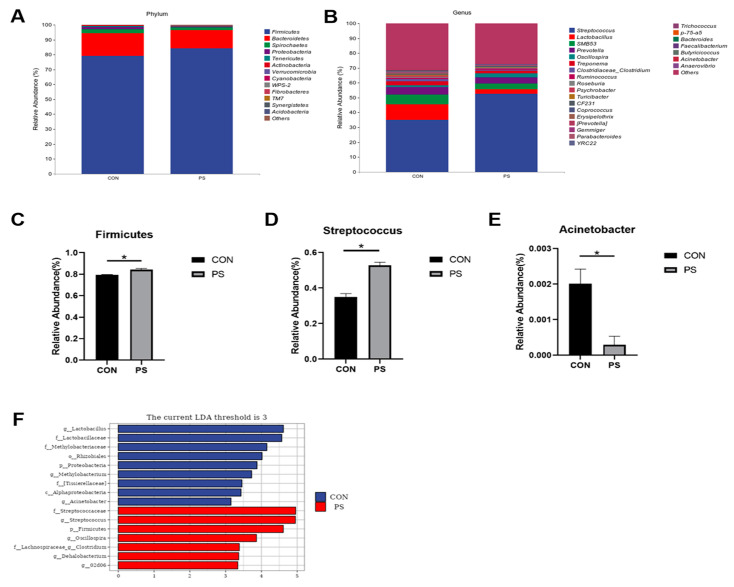
Effects of phytosterols (PSs) on the regulation of fecal microbiota in finishing pigs. (**A**) Phylum-level taxonomic structure. (**B**) Genus-level taxonomic structure. (**C**) Proportional representation of *Firmicutes*. (**D**) Proportional representation of *Streptococcus*. (**E**) Proportional representation of *Acinetobacter*. (**F**) linear discriminant analysis (LDA). The CON group was given a standard diet, while the PS group was offered a diet fortified with 300 mg/kg of phytosterols (PSs). Data are presented as means ± SEM (*n* = 4). * indicates *p* < 0.05.

Furthermore, a taxonomic composition analysis was conducted to explore the fecal microbial communities, focusing on the top 13 phyla and the top 25 genus (Figure 2A,B). PSs considerably elevated the number of *Firmicutes* in finishing pig feces at the phyla level (*p* < 0.05, Figure 2C). The addition of PSs to the food considerably enhanced *Streptococcus* abundance in the genus-level analysis (*p* < 0.05, Figure 2D). Moreover, the PS group had a considerably reduced abundance of *Acinetobacter* (*p* < 0.05, Figure 2E).

Additionally, Linear Discriminant Analysis Effect Size (LEfSe) was employed to identify unique bacterial taxa between the two groups (*p* < 0.05, LDA score > 3, Figure 2F). Our findings indicated that dietary PS supplementation raised the relative concentration of *Streptococcaceae*, *Streptococcus*, *Firmicutes*, *Oscillospira*, *Clostridium*, *Dehalobacterium*, and *02d06* in the feces. However, PS supplementation decreased the relative abundance of *Lactobacillus*, *Lactobacillaceae*, *Methylobacteriaceae*, *Rhizobiales*, *Proteobacteria*, *Methylobacterium*, *Tissierellaceae*, *Alphaproteobacteria*, and *Acinetobacter* in the feces.

### 3.8. Spearman Correlation of Fecal Microbiota Versus Environmental Factors

**Figure 3:**
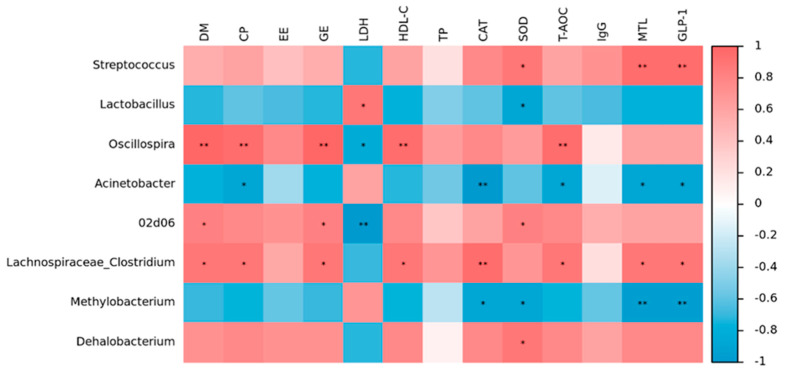
Heatmap: Spearman correlation of fecal microbiota versus phenotypes. * indicates *p* < 0.05. ** indicates *p* < 0.01.

At the genus level, correlation analysis was performed between some physiological and biochemical indices and fecal flora of the two groups of finishing pigs. *Streptococcus* showed positive correlations to SOD, MTL, and GLP-1 concentrations. *Lactobacillus* levels showed a positive association with LDH content, yet an inverse relationship with SOD activity. *Oscillospira*’s relative abundance had a negative correlation with LDH content, but a positive correlation with DM, CP, GE, HDLC, and T-AOC contents. Inverse relationships existed between *Acinetobacter* and CP, CAT, T-AOC, MTL, and GLP-1 levels, but a positive correlation with the amount of GAS was found. The proportion of *02d06* was higher when there were more DM, GE, and SOD, but lower when there was more LDH. Similarly, a higher proportion of *Clostridium* was linked to greater amounts of DM, CP, GE, HDL-C, CAT, T-AOC, MTL, and GLP-1, but less GAS. *Methylobacterium* levels showed inverse relationships with CAT, SOD, MLT, and GLP-1 levels, but a direct relationship with GAS concentration. The concentration of Dehalobacterium indicated a positive correlation with the SOD level (Figure 3).

## 4. Discussion

Several researches showed that PSs significantly improve animals’ growth performance and feed conversion rate [15,24,25]. Previous research pointed out that in the experiment of feeding mice with 89 mg/kg PS, the ADFI and the growth performance of mice were significantly improved [26]. Combined with our findings from this research, PSs significantly improved the ADFI of finishing pigs, which may be due to the fact that PSs promoted the release of gastrointestinal hormones, improved gastrointestinal motility, and increased appetite [27]. These factors contributed to the improvement of growth performance of finishing pigs to a certain extent. Additionally, broiler ADG and feed conversion were significantly boosted by adding 25 mg/kg of PSs to the diet [28]. Adding phytosterol ester to the diet can effectively reduce F:G [29]. However, no changes in weight gain or feed conversion rate were observed in our research upon the administration of PSs. This inconsistency with the conclusions of this experiment might stem from the possibility that PS additives trigger a surge in GLP-1 secretion, which, in turn, could lead to a hypoglycemic effect, putting the brakes on significant weight gain in those finishing pigs. In addition, the variations in PS treatment during animal experiments, along with differences in animal species, feeding habitats, feeding methods, and management practices, could also account for the discrepancies in the conclusions drawn from prior studies [28].

Apparent digestibility of nutrients is an important index for determining the growth performance of pigs [30]. Dietary PS supplementation significantly impacts nutrient digestion, absorption, and metabolism. Studies have reported that β-sitosterol, α-sitosterol, and dehydroergosterol can change the composition of intestinal microbiota, regulate gastrointestinal hormones, enhance intestinal barrier function, and improve apparent digestibility of nutrients in pigs [31]. In this experiment, the apparent digestibility of DM, CP, GE, and EE in pigs was significantly improved by adding PSs. This suggests that PSs may ultimately improve the growth and development of finishing pigs by improving digestibility and absorption of feed nutrients and promoting gastrointestinal function [32].

Our findings revealed that the serum concentrations of HDL-C and TP increased, while the LDH concentration decreased. PSs may modulate cellular signaling pathways by interacting with membrane-bound receptors, thereby regulating lipid metabolism and cellular function [33]. HDL-C facilitates the transport of macromolecular lipids, such as cholesterol, to the liver for decomposition [34], effectively improving the efficiency of lipid metabolism and providing protection against oxidative stress. Randomized controlled trials of PS treatment for cardiovascular damage were included in a meta-analysis, and these findings indicated that PS intake can significantly increase HDL-C levels in serum [25,35]. This is consistent with our findings. TP, BUN, ALB, and GLB in serum can reflect the absorption and metabolism of protein. Elevated TP levels indicate that PSs improve protein anabolism, thereby regulating the growth of finishing pigs. Research has demonstrated that optimal levels of PSs can effectively modulate protein metabolism by regulating both anabolic and catabolic processes, and affect cell proliferation and growth capabilities [36]. Furthermore, PSs can also interact with enzymatic systems to alter catalytic activity, thereby affecting the catalytic process of enzymes and playing a role in cellular metabolism and the body’s physiological processes [37]. Previous findings reported that PSs can promote protein anabolism and anti-inflammatory influence, accelerate the growth and development of poultry, and improve meat quality [38]. LDH can catalyze the conversion of pyruvate to lactate or vice versa [39]. As one of the biological indicators of immunosuppression, elevated LDH correlates with immunosuppressive cell activity [40,41], while the decrease in LDH may have a potential protective effect against inflammation in the body, as shown in our study.

Antioxidant capacity serves as a crucial physiological parameter in finishing pigs, exerting significant influence on growth performance, while maintaining substantial associations with developmental processes, inflammatory regulation, and immune system modulation [42]. ROS are oxygen-derived molecules primarily produced by NO and NADPH oxidase, which can cause cellular damage [43]. Studies reported that PSs may protect cells from oxidative damage by increasing antioxidant enzyme activity and inhibiting selected proteins in the redox signaling pathway [27], thereby effectively interfering with ROS [20]. Zhao et al. reported that PSs increase the activity of SOD in Partridge Shank chickens [44], which is consistent with our research findings.

In this study, it was found that feeding PSs improved the immune function of finishing pigs. This was reflected in the increase in IgG cytokine levels. B cell-produced IgG combats bacterial/viral invasions, providing immune defense. According to previous studies, PSs can effectively improve the immunity of mice. Serum ALB and IgA concentrations in mice rose notably, and the effect of PSs on increasing ALB levels was better than that of phytosterol ester [26]. The levels of CXCL1 and CXCL2 were markedly decreased. CXCL chemokines are secreted by monocytes and macrophages and have chemotactic effects on polymorphonuclear leukocytes and hematopoietic stem cells, which may indicate that PSs have potential anti-inflammatory and anti-tumor effects [45]. According to Yuan et al., dietary supplementation with PSs of 20 mg/kg and 40 mg/kg increased IgA and IgG concentrations [46]. Similarly, it was reported that PS additives increased the TP and IgG content in piglets [47]. These findings are consistent with our study. The benefits of PSs in regulating the immune system and improving disease resistance in animals may be attributed to the increase in the apparent digestibility of nutrients such as protein, the raise in the secretion of growth-related hormones, and the promotion of protein anabolism, resulting in elevated immunoglobulin levels [48].

To further explore the effect of PSs on the digestive capacity of finishing pigs, we examined the levels of gastrointestinal hormones in serum. The levels of MTL and GLP-1 were both increased. MTL is a hormone mainly released by the small intestine to promote gastric emptying [49,50]. It has been shown that PSs can regulate gastrointestinal motility, and satiety signals. When MTL levels are elevated [51], the stomach releases gastric acid to help digest food [52]. In addition, it was reported that feeding rats with synthetic gastrin analogs can increase their protein synthesis [53]. Since MTL has a certain nutritional effect on the gastrointestinal mucosa [54], we speculate that this could alter the gut milieu, favorably impacting colonization by beneficial microbes and diversifying gut flora. The gastrointestinal hormone GLP-1 released by intestinal L-type cells can promote fatty acid oxidation [55] and improve glucose metabolism in the liver to reduce lipogenesis [51]. Numerous studies found that mixtures containing PSs can increase GLP-1 levels [56,57].

Our research examined the impact of PSs on fecal microbiota using 16S rRNA sequencing. Studies on α and β diversity revealed that dietary PS supplementation can increase the diversity of fecal microbiota. The lower Simpson index denotes heightened interspecies variation, reflecting elevated biodiversity. Simpson’s evenness index can provide a more intuitive understanding of the biodiversity level in a community. As depicted in the Venn diagram, the PS group exhibited more unique ASVs than the CON group. At the phylum and genus levels, the enrichment of Firmicutes in the PS group was observed. It is worth noting that certain members of Firmicutes churn out SCFAs (short-chain fatty acids), regulating intestinal immunity [11]. Some reports have revealed that certain beneficial bacteria of *Streptococcus* (such as *Streptococcus* thermophilus) can effectively inhibit the proliferation of pathogens and improve gastrointestinal function [58]. *Acinetobacter* is prone to infection in the body [59], and PS supplementation reduces the relative abundance of *Acinetobacter*.

Through further analysis of LEfSE, at the genus level, we found that *Streptococcus*, *Oscillospira*, *Lachnospiraceae clostridium*, and *Dehalobacterium* were significantly enriched in the PS group. In this study, it was also found that *Oscillospira* was positively correlated with the digestibility of DM, CP, and GE. Some previous reports have found that *Oscillospira* can produce butyrate [60], which promotes appetite, aids digestion, and thus protects intestinal function. *Clostridium* can increase the number of Treg cells in adult mice, thereby improving resistance to intestinal pathogens and the ability to maintain autoimmune homeostasis [61]. *Dehalobacterium* can help decompose toxic substances [62] and reduce the accumulation of harmful substances [63], potentially leading to significant improvements in intestinal microbial homeostasis and overall gut ecosystem balance. In addition, some findings have shown there is a potential negative correlation between *Dehalobacterium* and the development of inflammation [64,65]. We conclude that PS supplementation-induced changes in gut bacteria such as *Streptococcus*, *Oscillospira,* and *Dehalobacterium* may have led to improved immunity, antioxidant levels, and nutrient digestion in finishing pigs. More research is required to determine the precise mechanisms behind these relationships.

## 5. Conclusions

In conclusion, phytosterols hold great potential as a natural additive in feed. Dietary phytosterol supplementation may improve growth performance by enhancing nutrient digestibility and promoting the secretion of gastrointestinal hormones. It has beneficial effects on improving the antioxidant and immune capacity of animals. Phytosterols can also change the fecal microbiota in finishing pigs. Phytosterols up-regulated the abundance of *Firmicutes* and *Streptococcus* and down-regulated the abundance of *Acinetobacter*.

## Figures and Tables

**Table 1:** Feedstuff composition and nutritional content.

Items	Content
Ingredients	
Corn	73.00
Soybean meal (%)	18.00
Wheat bran (%)	5.00
Lysine (%)	0.30
CaHPO_4_ (%)	0.50
Limestone (%)	1.09
NaCl (%)	0.40
Phytase 5000 IU (%)	0.04
Premix (%)	1.67
Total	100.00
Nutrient levels (%)	
DE (MJ/kg) (%)	14.20
Crude protein (%)	14.31
Total Lysine (%)	1.03
Ca (%)	0.62
TP (%)	0.41

The premix contained the following vitamins and minerals per kg: VA at 15,000 IU, VD3 at 3100 IU, VE at 160 IU, VK3 at 3 mg, VB1 at 3 mg, VB2 at 6 mg, VB6 at 5 mg, VB12 at 0.03 mg, VC at 250 mg, D-pantothenic acid at 9 mg, niacin at 45 mg, biotin at 0.3 mg, folic acid at 1 mg, iron (FeSO_4_·H_2_O) at 170 mg, copper (CuSO_4_·5H_2_O) at 15 mg, manganese (MnSO_4_·H_2_O) at 80 mg, zinc (ZnSO_4_·H_2_O) at 150 mg, iodine (KI) at 0.9 mg, selenium (Na_2_SeO_3_) at 0.2 mg, magnesium (MgO) at 69 mg, and cobalt (CoCl_2_) at 0.3 mg. The digestibility energy (DE) was an estimated figure, while the rest of the values were obtained through direct measurement.

**Table 2:** The effect of phytosterols (PSs) on growth performance in finishing pigs.

Items	CON	PS	*p*-Value
Initial BW, kg	79.4 ± 1.6	80.36 ± 2.4	0.763
Final BW, kg	111.43 ± 1.32	113.58 ± 0.96	0.217
ADG, kg	0.94 ± 0.06	0.98 ± 0.04	0.631
ADFI, kg	2.44 ± 0.02	2.56 ± 0.03 *	0.012
F:G	2.62 ± 0.18	2.64 ± 0.11	0.919

The CON group was given a standard diet, while the PS group was offered a diet fortified with 300 mg/kg of phytosterols (PSs). IBW for initial body weight; FBW for final body weight; ADG for average daily gain; ADFI for average daily feed intake; F:G for feed-to-gain ratio. Data are presented as means ± SEM (*n* = 5). * indicates *p* < 0.05.

**Table 3:** The effects of phytosterols (PSs) on nutrient apparent digestibility in finishing pigs.

Items	CON	PS	*p*-Value
DM	89.18 ± 0.4	93.64 ± 0.21 *	<0.01
CP	87.95 ± 1.05	91.61 ± 0.5 *	0.014
EE	77.2 ± 3.16	86.18 ± 1.34 *	0.031
GE	89.69 ± 0.44	93.9 ± 0.26 *	<0.01
CF	63.13 ± 1.89	63.73 ± 2.63	0.859

The CON group was given a standard diet, while the PS group was offered a diet fortified with 300 mg/kg of phytosterols (PSs). DM for dry matter; CP for crude protein; EE for ether extract; GE for gross energy; CF for crude fiber. Data are presented as means ± SEM (*n* = 5). * indicates *p* < 0.05.

**Table 4:** The effect of phytosterols (PSs) on serum biochemical indicators in finishing pigs.

Items	CON	PS	*p*-Value
ALT (U/L)	77.92 ± 9.26	72.97 ± 9.42	0.723
ALP (U/L)	179.07 ± 14.43	195.95 ± 27.66	0.603
AST (U/L)	32.86 ± 2.13	30.99 ± 2.36	0.586
LDH (U/L)	711.68 ± 29.74	522.2 ± 42.53 *	<0.01
HDL-C (mmol/L)	1.17 ± 0.04	1.3 ± 0.01 *	0.013
LDL-C (mmol/L)	1.22 ± 0.03	1.13 ± 0.05	0.175
BUN (mmol/L)	4.64 ± 0.67	6.32 ± 0.48	0.074
GLU (mmol/L)	4.09 ± 0.11	5.28 ± 0.47	0.066
TC (mmol/L)	2.61 ± 0.18	3.08 ± 0.16	0.096
TG (mmol/L)	0.51 ± 0.01	0.64 ± 0.21	0.570
TP (g/L)	71.25 ± 4.31	85.05 ± 3.76 *	0.042
ALB (g/L)	33.66 ± 4.87	47.69 ± 4.59	0.069
GLB (g/L)	37 ± 0.41	37.2 ± 1.07	0.879

The CON group was given a standard diet, while the PS group was offered a diet fortified with 300 mg/kg of phytosterols (PSs). ALT for alanine aminotransferase; ALP for alkaline phosphatase; AST for aspartate aminotransferase; LDH for lactate dehydrogenase; HDL-C for high-density lipoprotein cholesterol; LDL-C for low-density lipoprotein cholesterol; BUN for blood urea nitrogen; GLU for glucose; TC for total cholesterol; TG for triglyceride; TP for total protein; ALB for albumin; GLB for globulin. Data are presented as means ± SEM (*n* = 5). * indicates *p* < 0.05.

**Table 5:** The effect of phytosterols (PSs) on antioxidant indicators in finishing pigs.

Items	CON	PS	*p*-Value
GSH-Px (μmol/L)	1463.21 ± 168.47	1659.37 ± 44.04	0.200
MDA (nmol/mL)	3.45 ± 0.54	2.89 ± 0.26	0.402
GSH (mg/L)	7.58 ± 0.55	12.29 ± 1.89	0.094
CAT (U/Ml)	6.29 ± 0.45	8.81 ± 0.23 *	<0.01
SOD (U/mL)	29.98 ± 3.27	49.75 ± 3.83 *	<0.01
T-AOC (U/mL)	0.84 ± 0.05	1.09 ± 0.05 *	0.012

The CON group was given a standard diet, while the PS group was offered a diet fortified with 300 mg/kg of phytosterols (PSs). GSH-Px for glutathione peroxidase; MDA for malondialdehyde; GSH for glutathione; CAT for catalase; SOD for superoxide dismutase; T-AOC for total antioxidant capacity. Data are presented as means ± SEM (*n* = 5). * indicates *p* < 0.05.

**Table 6:** The effect of phytosterols (PSs) on the production of immune cytokines in finishing pigs.

Items	CON	PS	*p*-Value
IL-2 (ng/mg)	483.78 ± 21.71	479.13 ± 34.57	0.912
IL-4 (ng/mg)	66.23 ± 4.23	66.97 ± 4.03	0.903
IL-6 (ng/mg)	872.84 ± 82.1	882.55 ± 88.22	0.939
TNF-α (pg/mg)	386.64 ± 35.56	457.14 ± 33.36	0.186
IgA (g/L)	32.81 ± 2.59	34.54 ± 2.78	0.670
IgM (g/L)	31.33 ± 2.71	34.65 ± 1.75	0.319
IgG (g/L)	346.89 ± 14.78	399.2 ± 16.63 *	0.047

The CON group was given a standard diet, while the PS group was offered a diet fortified with 300 mg/kg of phytosterols (PSs). IL-2 for interleukin 2; IL-4 for interleukin 4; IL-6 for interleukin 6; TNF-α for tumor necrosis factor-α; IgA for immunoglobulin A; IgM for immunoglobulin M; IgG for immunoglobulin G. Data are presented as means ± SEM (*n* = 5). * indicates *p* < 0.05.

**Table 7:** The effect of phytosterols (PSs) on intestinal hormone levels in serum.

Items	CON	PS	*p*-Value
GAS (pg/mL)	171.45 ± 5.86	151.11 ± 8.09	0.076
MTL (pg/mL)	331.23 ± 8.07	424.56 ± 20.3 *	<0.01
GLP-1 (pmol/L)	3.03 ± 0.13	3.92 ± 0.15 *	<0.01

The CON group was given a standard diet, while the PS group was offered a diet fortified with 300 mg/kg of phytosterols (PSs). GAS for gastrin; MTL for motilin; GLP-1 for glucagon-like peptide-1. Data are presented as means ± SEM (*n* = 5). * indicates *p* < 0.05.

## Data Availability

The raw data presented in this study are available upon request from the respective authors.
